# Sterculic Acid Alters Adhesion Molecules Expression and Extracellular Matrix Compounds to Regulate Migration of Lung Cancer Cells

**DOI:** 10.3390/cancers13174370

**Published:** 2021-08-29

**Authors:** Rafael Peláez, Rodrigo Ochoa, Ana Pariente, Ángela Villanueva-Martínez, Álvaro Pérez-Sala, Ignacio M. Larráyoz

**Affiliations:** Biomarkers and Molecular Signaling Group, Neurodegeneration Area, Center for Biomedical Research of La Rioja (CIBIR), Piqueras 98, 26006 Logroño, Spain; rochoaf@riojasalud.es (R.O.); apariente@riojasalud.es (A.P.); angela.v.m.1997@outlook.es (Á.V.-M.); aperez@riojasalud.es (Á.P.-S.)

**Keywords:** sterculic acid, extracellular matrix, cell migration, gene expression

## Abstract

**Simple Summary:**

Sterculic acid (SA) is a naturally occurring lipid with SCD1 inhibitory activity, but it also modifies many other pathways and underlying gene expression. *SCD* upregulation has been associated with tumor aggressiveness and progression. Effects of SA treatment over extracellular matrix compounds and adhesion molecule expression have not been described in cancer cells up to now. Our results show that SA induces cell death at high dose, but we also observed that lower concentrations of SA treatments also reduce cell adhesion-migration and modify integrins and extracellular matrix compounds expression.

**Abstract:**

Sterculic acid (SA) is a cyclopropenoid fatty acid isolated from *Sterculia foetida* seeds. This molecule is a well-known inhibitor of SCD1 enzyme, also known as ∆9-desaturase, which main function is related to lipid metabolism. However, recent studies have demonstrated that it also modifies many other pathways and the underlying gene expression. *SCD* overexpression, or up-regulated activity, has been associated with tumor aggressiveness and poor prognosis in many cancer types. Scd1 down-regulation, with different inhibitors or molecular strategies, reduces tumor cell survival and cell proliferation, as well as the chemoresistance associated with cancer stem cell presence. However, SA effects over cancer cell migration and extracellular matrix or adhesion molecules have not been described in cancer cells up to now. We used different migration assays and qPCR gene expression analysis to evaluate the effects of SA treatment in cancer cells. The results reveal that SA induces tumoral cell death at high doses, but we also observed that lower SA-treatments induce cell adhesion-migration capacity reduction as a result of modifications in the expression of genes related to integrins and extracellular matrix compounds. Overall, the functional and transcriptomic findings suggest that SA could represent a new inhibitor activity of epithelial to mesenchymal transition.

## 1. Introduction

Our understanding of the role of the tumor microenvironment (TME) over the recurrence and relapse of cancer has been growing in the last years. The physical properties and composition of the TME generate signals to alter cancer cell proliferation and migration [1,2] and it is thought that the TME could represent a protective niche for tumoral cells during antitumoral therapies [3,4]. The extracellular matrix (ECM) is one of the most relevant elements that compose the TME [5] and it represents a scaffold structure for cells. The ECM is composed of many proteoglycans, glycoproteins and fibrous proteins [6,7,8] and the organization and composition of this matrix confers each tissue its own characteristics [8,9]. ECM compounds are modified by tumor cells directly, or in an indirect way, by normal cells to promote cancer cell proliferation, survival or metastasis [10,11].

Collagen is the main structural constituent of the ECM and its aberrant expression, deposition, alignment or cross-linking has been correlated with epithelial-mesenchymal (EMT), tumor metastasis and drug resistance [12]. Collagen fibers and other glycoproteins, such as fibronectin, laminin or elastin, form a mesh that interacts with cells in an adhesion receptor-dependent manner [13,14]. PGs and glycosaminoglycans (GAGs) are also presents in the mesh in the interstitial spaces of the ECM, to capture and store biomolecules and growth factors which regulate cell proliferation, migration and differentiation [15]. The high complexity of the ECM and its alterations promote remarkable changes in matrix properties that are linked to different diseases [16]. Cell–cell and cell–matrix interactions modulate cancer progression and development [17]. The ECM represents a scaffold support for cells, but it also activates signal pathways mediated by membrane receptors that modulate cancer cell hallmarks. Cells are constantly synthesizing, destroying and modifying their ECM. Increased collagen fibers deposition has been also associated with tumor progression and advanced stages of cancer [18]. It has been demonstrated that matrix density blocks antitumoral drug access to the intratumoral cells [19] but it has also been observed that matrix remodeling is associated with tumor progression [20].

Sterculic acid (SA) is a natural lipid present in multiple plant seeds and it is the main component of *Sterculia foetia* seed oil [21]. This lipid has been described as an inhibitor of the stearoyl–CoA desaturase (SCD) protein and the subsequent transformation of stearic acid to oleic acid [22]. SA treatment reduces monounsaturated fatty acids (MUFAs) levels and has positive effects over pathologies such as glucose tolerance, blood pressure and obesity [23,24,25,26,27]. SCDs overexpression has been observed in many cancer types and it is associated with tumor aggressiveness, poor prognosis and reduction of relapse-free survival of patients of breast cancer and hepatocellular carcinoma (HCC) [28]. SCD1 activity increases membrane MUFAs to promote cell viability [29]. SCD1 inhibition reduces the proliferation of prostate and lung cancer cells [30], and induce cell death [28,31]. Recent studies have demonstrated that SA neutralizes the 7-ketocholesterol (7Kch) induced cytotoxicity in vitro and in vivo models of choroidal neovascularization (CNV) [32]. Molecular mechanisms underlying the SA beneficial effects are still unknown. SA administration modify lipogenic genes such as ACC, FAS, SREBP1a/c [24,33], but it also activate mechanisms against cell injuries such as C/EBP homologous protein (CHOP), glucose-regulated protein, 78 KDa (GRP78) [32] mediated by TLR4 and the activation of many intracellular kinases [34]. However, a transcriptomic analysis of SA treatment of retinal pigmented epithelium (RPE) cells has revealed that this lipid induces a wide range of genomic modifications that affects ECM molecule secretion (COL1A1 and CAV1), cell adhesion (ITGα5), metabolism (ACC1, SREBF1, APOE) and angiogenesis (ANGPTL4 and PDGFB) pathways in a SCD1-independent manner [35]. 

The effects of SA treatment over tumor cells have not been described until now. In the present work we reveal that SA induces tumor cell death in a time- and dose-dependent manner, which is also mediated, at least in part, by a Caspase-3 activation. Our results also demonstrate that lower SA treatments reduce cell wound healing and migration capacity and modify the expression of genes related to cell adhesion an extracellular matrix compounds.

## 2. Materials and Methods

### 2.1. Cell Lines and Culture

A549 and H1299 cells are non-small lung cancer cells obtained from the ATCC (Manassas, VA 20108, USA). A549 is a human lung carcinoma cell line isolated from a 58-year-old male. It presents an epithelial morphology with adherent capability. H1299 is also a human lung carcinoma cell line isolated from a 43-year-old male. It presents an epithelial morphology with adherent capacity. H1299 and A549 cell lines were cultured in RPMI 1640 medium (Hyclone-Thermo Scientific, Waltham, MA, USA) supplemented with 10% fetal bovine serum (Invitrogen, Alcobendas, Madrid, Spain) and 1% penicillin/streptomycin (Hyclone-Thermo Scientific). Cells were grown in a 37 °C environment, with an atmosphere containing 5% CO_2_ and 85% humidity.

### 2.2. Cell Treatments

Cells were seeded at a density of 25,000 cells/well or 50,000 cells/well, in serum and serum free conditions, respectively, in 48-well plates for MTT assays. Serum depleted media was composed of RMPI1640 and 1% penicillin/streptomycin. A total of 350,000 cells were seeded in 6-well plates for migration and wound healing assays. Cells attached for 24 h and grew until 100% confluence. Then, serum-containing media was removed and was changed for serum-free medium for 24 h before treatment administration. Cells were treated with sterculic acid (SA) solved in DMSO from 0 to 250 µM (PPQF, University of Alcalá, Madrid, Spain) for 24 to 72 h.

### 2.3. Cell Viability Assay

Cell viability was analyzed after 24 h using the 3-(4,5-dimethyl thiazol-2-yl)-5-(3-carboxymethoxyphenyl)-2-(4-sulfophenyl)-2H-tetrazolium bromide (MTS) assay (Promega, Madison, WI, USA). Two washes with PBS were made before 1:10 MTS-medium mixture adding. Cell viability was measured after 2 h of incubation at 37 °C. Results are presented as viability over vehicle-treated cells.

### 2.4. Wound Healing Assay

The monolayer was scratched using a pipette tip and we captured images at the same location at regular intervals (24 h) during cell migration to close the scratch using a Leica Inverted Microscope and Leica DMI4000B inverted microscope (Leica Microsystems), with N Plan 2.5-20X objectives and equipped with a Leica DFC300 Fx digital camera and using Leica Application Suite V3.3.0. The healed surfaces were further analyzed and quantified using Fiji computing software. 

### 2.5. Transwell Migration Assay

Cells were harvested 24 h after treatment administration and 40,000 cells were plated onto the upper side of 8-μm pore-size Boyden chamber (Corning) previously pre-coated with type I rat tail collagen. Cells were cultured in serum-free media for 2 h before allowing cell migration towards complete media at 37 °C for 18–24 h. Cells were fixed in 4% formaldehyde for 15 min and washed two times with PBS. The Boyden chamber upper part, where the cells had been seeded, was completely cleaned with cotton swabs before the inserts were stained with 0.5% crystal violet. Images from the lower part of Boyden chamber were captured using a Leica DMI4000B inverted microscope (Leica Microsystems), with N Plan 2.5-20X objectives and equipped with a Leica DFC300 Fx digital camera and using Leica Application Suite V3.3.0. The number of cells in the lower side of the membrane was counted on images taken from six random fields per transwell.

### 2.6. RNA Purification

TRIzol (Invitrogen, Madrid, Spain) was used in total RNA isolation from cell cultures and it was purified using the RNeasy mini-kit (Qiagen, Valencia, CA, USA). DNase I (Qiagen, Valencia, CA, USA) was used to treat the samples following the manufacturer’s instructions.

### 2.7. Quantitative Real-Time PCR

SuperScript III kit (Invitrogen, Madrid, Spain) and random primers were used to reverse-transcribe 1 µg of total RNA into first-strand cDNA in a total volume of 20 µL according to the manufacturer’s instructions. cDNA was mixed with SYBR Green PCR Master Mix (Applied Biosystems, Carlsbad, CA, USA) for quantitative real time polymerase chain reaction (qRT-PCR) using 0.3 µM forward and reverse oligonucleotide primers (Table 1). A 7300 Real Time PCR System (Applied Biosystems, Madrid, Spain) was employed in the quantitative measures. Cycling conditions were: an initial denaturation at 95 °C for 10 min, followed by 40 cycles of 95 °C for 15 s and 60 °C for 1 min. At the end, a dissociation curve was implemented from 60 to 95 °C to validate amplification specificity. Gene expression was calculated using absolute quantification by interpolation into a standard curve. 18S gene expression was used as housekeeping.

### 2.8. Western Blotting

Western blot was performed as described previously [36]. Cells were scraped and homogenized in RIPA buffer (Thermo Scientific) containing protease (EDTA-free complete, Roche, Basilea, Switzerland) and phosphatase inhibitors (PhosStop, Roche, Basilea, Switzerland). Homogenates were centrifuged for 30 min at 15,000× *g* and the supernatants collected. Protein concentration was determined by the BCA kit (Pierce, Rockford, IL, USA), with bovine serum albumin as standard, using a spectrophotometer POLARstar Omega, (BMG Labtech, Ortenberg, Germany). Then, 20 µg of each sample were mixed with 4x Sample Buffer (Invitrogen, Madrid, Spain) and heated for 10 min at 70 °C. Samples were run on 4–12% SDS–polyacrylamide gels. SeeBlue plus 2 pre-stained standards (Invitrogen) were used as molecular weight markers. For Western blot analysis, proteins were transferred onto 0.2-μm polyvinylidene difluoride (PVDF) membranes (iBlot system, Invitrogen). For protein identification, membranes were incubated overnight at 4 °C with a rabbit polyclonal antibody against Caspase-3 (14220S, Cell Signaling, Danvers, MA, USA) at a dilution 1:1000. To standardize the results, a monoclonal IgG anti-β-Actin antibody (Sigma-Aldridch, Steinheim, Germany) was used at a dilution 1:5000 in the same membranes. To visualize immunoreactivity, membranes were incubated with anti-mouse (715-035-1514, Jackson Immunoreserarch Lab. West Grobe, PA, USA) and anti-rabbit (7074, Cell Signaling) peroxidase-labeled IgGs, and they were developed with a chemiluminescence kit ClarityTM Western ECL (BIO-RAD, Berkeley, CA, USA), and exposed to X-ray films (Amersham Hyperfilm ECL, GE Healthcare, Buckinghamshire, UK).

### 2.9. Statistical Analysis

All data were analyzed with GraphPad Prism 6 software and were considered statistically significant when *p*-value < 0.05. Values are expressed as means ± SEM. Non-normal distributed data were evaluated by Mann–Whitney U test. Two-way ANOVA was used for multiple comparisons of independent means with Sidak modification to compare related samples.

## 3. Results

### 3.1. SA Induces Dose-Dependent Cytotoxicity in Cancer Cell Lines in Serum and Serum-Free Conditions

**Figure 1:**
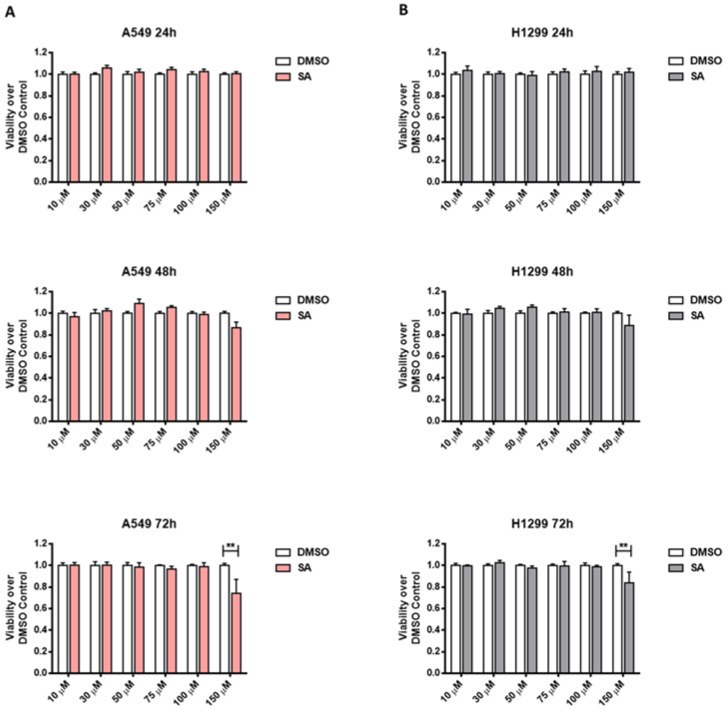
Sterculic acid induces cell death in a dose-time dependent manner in lung cancer cells cultured in 10% serum-supplemented medium. Cell viability after SA treatment (10–150 µM) was measured by MTS method. (**A**) Cell viability over DMSO control of A549 cells. White bars correspond to cells treated with vehicle (DMSO) while pink bars correspond to cells treated with SA (10–150 µM). (**B**). Cell viability over DMSO control of H1299 cells. White bars correspond to cells treated vehicle (DMSO) while grey bars correspond to cells treated with SA (10–150 µM). Data represented mean ± SEM of 48-well plates of at least three different experiments. ** *p* < 0.01.

We treated cells with a broad range of SA doses in medium containing 10% serum, from 24 to 72 h. We observed that both cell lines did not present cell cytotoxicity as a result of SA treatments at concentrations lower than 150 μM (Figure 1). However, we detected cell cytotoxicity in prolonged treatments at higher concentrations (200 μM and 250 μM) (Figure S1). Furthermore, A549 cells showed slightly more sensibility than H1299 to SA treatments (Figure S1A,B). This increased effect was also observed in the non-tumorigenic human bronchial epithelial cell line, NL20 (Figure S2A).

**Figure 2:**
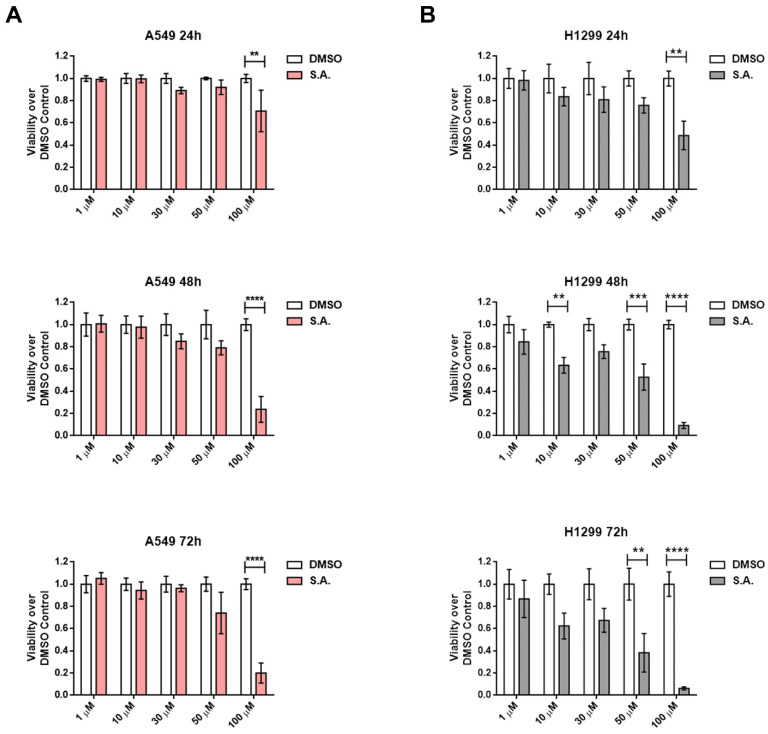
Sterculic acid induces cell death in a dose-time dependent manner in lung cancer cells cultured in serum-free medium. Cell viability after SA treatment (1–100 µM) was measured by MTS method. (**A**) Cell viability over DMSO control of A549 cells. White bars correspond to cells treated with vehicle (DMSO), while pink bars correspond to cells treated with SA (1–100 µM). (**B**) Cell viability over DMSO control of H1299 cells. White bars correspond to cells treated vehicle (DMSO) while grey bars correspond to cells treated with SA (1–100 µM). Data represented mean ± SEM of 48-well plates of at least three different experiments. ** *p* < 0.01, *** *p* < 0.001, **** *p* < 0.0001.

**Figure 3:**
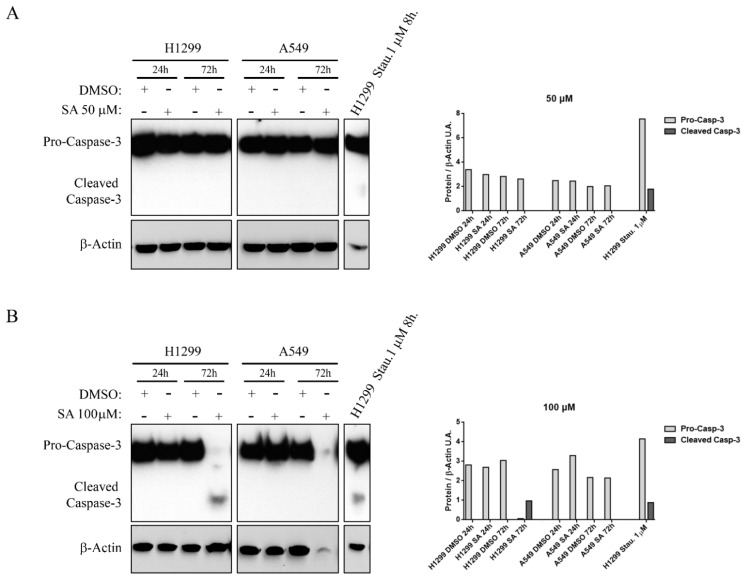
Caspase-3 apoptotic pathway participates in SA-induced cell death. Representative Western blot of Caspase-3 activation in protein extracts of H1299 and A549 cell lines and quantification of Western blot signal intensity (right graphics). Values represents the Pro-Caspase-3 or Cleaved Caspase-3 over β-Actin signal in UA. Staurosporine 1 µM treatment in H1299 for 8 h was used as positive control of Caspase-3 cleavage and apoptosis activation. (**A**) Caspase-3 levels after 24 and 72 h of 50 µM SA treatments. (**B**) Caspase-3 levels after at 24 and 72 h of 100 µM SA treatments.

In order to understand the cellular effects of SA without the interference of lipids of serum, we tested the hypothesis of a delayed effect of SA in serum using MTS assay in serum-free conditions. Figure 2 shows that low SA concentrations have no significant effects over cell viability, and only 100 μM SA presented clear cytotoxic effects in these cell lines. We could also observe that H1299 cell line showed a less stable response (Figure 2B) but more sensibility to SA treatments with low doses while A549 cell line is more resistant. In the non-tumorigenic cell line NL20, we also observed an intense cytotoxicity at high dose treatments in serum supplemented media (Figure S2A), while we detected a significant cytotoxicity at low doses around the 60% of the control cells in serum-free media (Figure S2B). Western blot analysis revealed that SA-induced cytotoxicity is associated with Caspase-3 activation in long term and high doses treatments (Figure 3B), while short term and lower doses did not show activation of this pro-apoptotic signaling (Figure 3A,B).

### 3.2. SA Reduces Cell Motility

Previous studies in retinal cells have demonstrated that low SA treatments promote the induction of transcriptional changes in multiple cellular pathways, and this could represent a new possible strategy against tumor cells [35]. We evaluated the effects of low dose treatments (1 µM, 10 µM and 30 µM) in cell migration capacity using a wound healing assay in serum-free media. This a basic assay to evaluate the cellular migration in vitro and it is particularly suitable for studies on the effects of cell–matrix and cell–cell interactions on cell migration.

**Figure 4:**
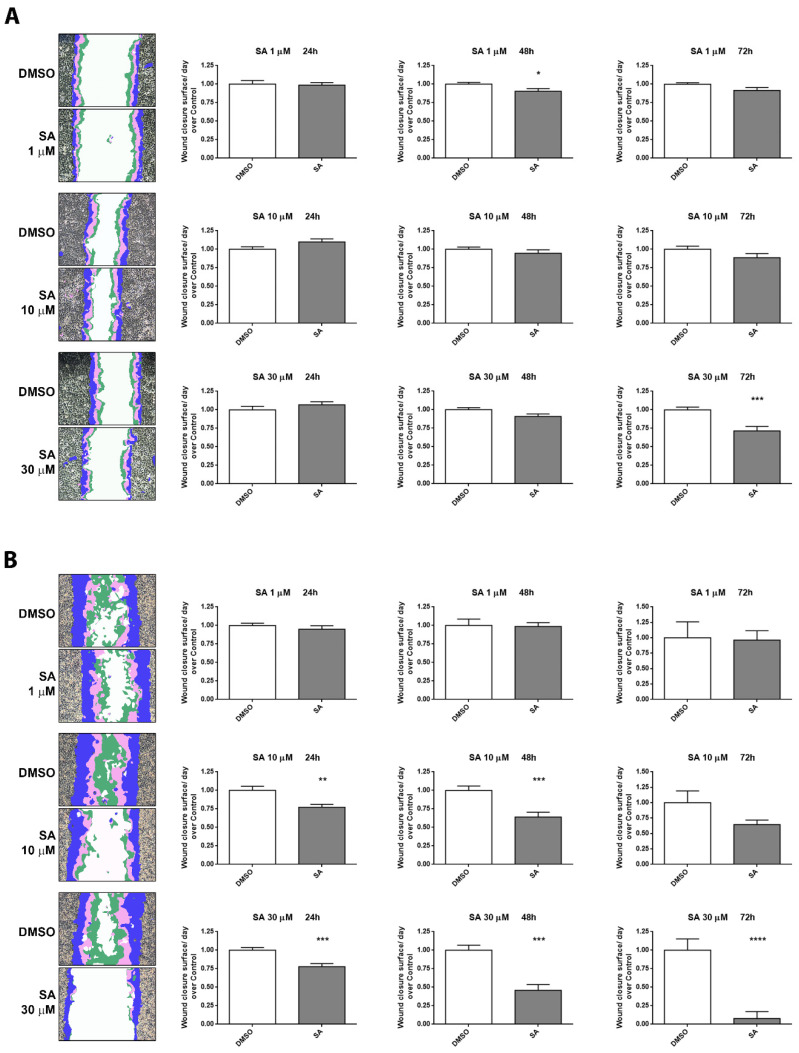
Sterculic acid disturbs wound healing in a dose-dependent manner. A549 (**A**) and H1299 (**B**) and cells were treated with SA at concentrations from 1 to 30 μM during 24 to 72 h in serum-free medium. Representative fields of wound healing area per day, where blue regions are the wound’s closed surfaces from 0 to 24 h, pink regions are the wound’s closed surface from 24 to 48 h, green regions are the wound’s closed areas from 48 to 72 h and white area represents the unclosed scratch region. In the graphs, white bars correspond to vehicle cell treatment (DMSO) while grey bars correspond to cells treated with SA (1–30 µM). Data represent mean ± SEM of closed area per day with respect to its DMSO control from at least two scratches of four independent biological replicates. * *p* < 0.05, ** *p* < 0.01, *** *p* < 0.001, **** *p* < 0.0001.

We observed that both cell lines tried to fill the wound although the migration capacity of A549 cells (Figure 4A) was lower than that of H1299 cells (Figure 4B). Nevertheless, a reduction of A549 cell migration was observed as a result of 30 µM SA treatment. Furthermore, a slight non-significant migration was also observed in A549 for other treatments (10 µM-72 h and 30 µM-48 h), (Figure 4A). Interestingly, a prominently SA-induced reduction of cell migration was observed in a time and dose dependent manner in H1299 cells. At 24 h, 30 µM SA treatments markedly reduced wound closure while they almost completely abrogated the migration after 72 h of treatment (Figure 4B). We also observed an important reduction in wound healing closure in H1299 cells, even with 10 µM treatments.

**Figure 5:**
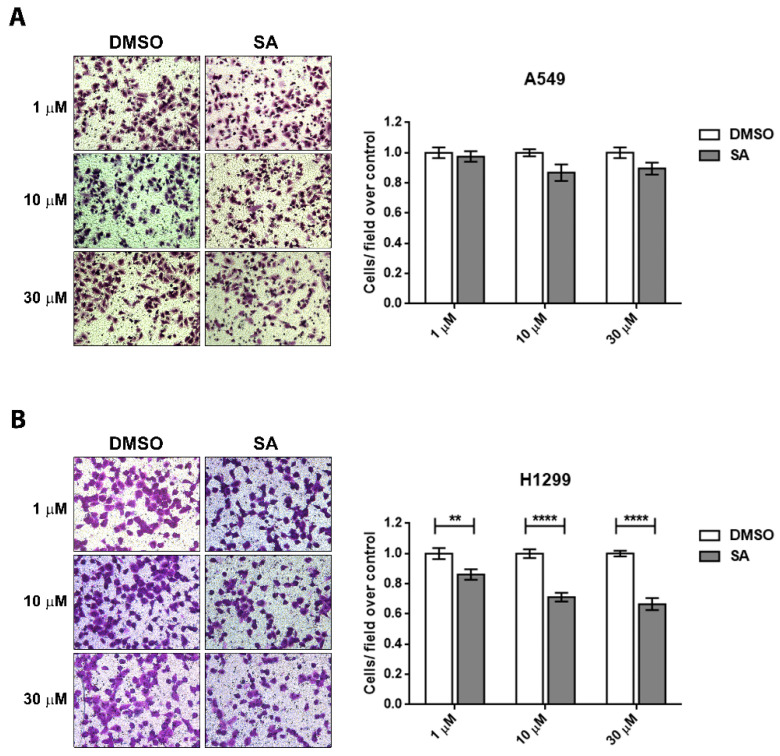
Sterculic acid reduces cell migration capacity in lung cancer cell lines. SA-treated A549 (**A**) and H1299 (**B**) cells were allowed to migrate across collagen-coated transwell chambers for 24 h. Representative fields of cell migration are included in figure. Data represents mean ± SEM of migrating cells with respect to its DMSO control from at least four independent biological replicates. White bars correspond to cells treated with vehicle (DMSO) while grey bars correspond to cells treated with SA (1–30 µM). ** *p* < 0.01, **** *p* < 0.0001.

We performed a Boyden chamber assay to specifically evaluate the migration capacity of these cells after SA treatment in serum-free conditions. In accordance with the wound healing assays, H1299 cell line presents a more intense migratory phenotype than A549. A clear cell-spreading morphology was observed on the lower side of the transwell’s membrane just after 18 h of migration, while A549 needed ≥24 h. A slight reduction of cell migration was detected in A549 cells when they were treated with 10 and 30 µM, although differences were not statistically significant (Figure 5A). However, all treatments tested were effective in the reduction of cell migration in H1299 cells (Figure 5B).

### 3.3. SA Modifies Expression of Cell Adhesion, Matrix Composition and Remodeling Genes

**Figure 6:**
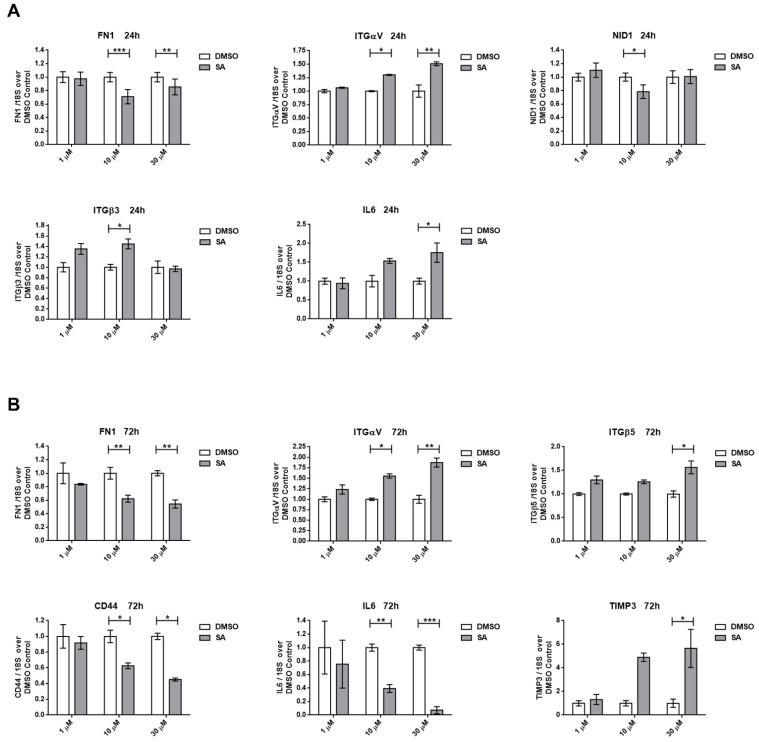
Altered gene expression signature of A549 cells after SA treatments. A broad panel of genes related to cell adhesion and extracellular matrix composition and remodeling was tested. (**A**) Significant gene expression of control and A549 with SA treated for 24 h. (**B**) Significant gene expression of control and A549 with SA treated for 72 h. Data represent mean ± SEM gene expression with respect to the 18S-housekeeping gene of three different experiments. * *p* < 0.05, ** *p* < 0.01, *** *p* < 0.001.

The ability of cells to migrate is a result of cell morphology changes, detachment modifications and/or matrix remodeling process, which are usually associated with gene expression modifications and protein activation/inactivation switches. As a consequence of the migration-observed changes after SA treatment, we decided to evaluate the expression of genes related to cell adhesion, matrix remodeling and ECM compounds. Figure 6 presents down-regulated and up-regulated genes in A549 cells as a result of SA treatment after 24 h or 72 h. Two genes of the ECM (FNI and NID1) were down-regulated, while adhesion molecules (ITGαV and ITGβ3) and inflammation-related genes (IL-6) were up-regulated after 24 h of treatment (Figure 6A). Other genes related to the ECM or cell–ECM interaction were assayed, although no significant expression changes were detected (Figure S3A).

In Figure 6B, we show the genes whose expression was altered after SA treatment after 72 h. ECM genes, such as FNI, had reduced expression, whereas TIMP3, a MMP inhibitor gene, was highly up-regulated. Adhesion molecules genes (ITGαV and ITGβ5) were upregulated, while other adhesion receptors, such as CD44, downregulated their expression.

IL-6 mRNA presented a markedly reduced expression. The same expression switch was detected for ITGβ3 at 30 µM SA treatment, but it was not statistically significant (Figure S4B). Finally, SCD expression was also upregulated in A549 cells after 24 and 72 h of SA treatments (Figure S5A,B).

**Figure 7:**
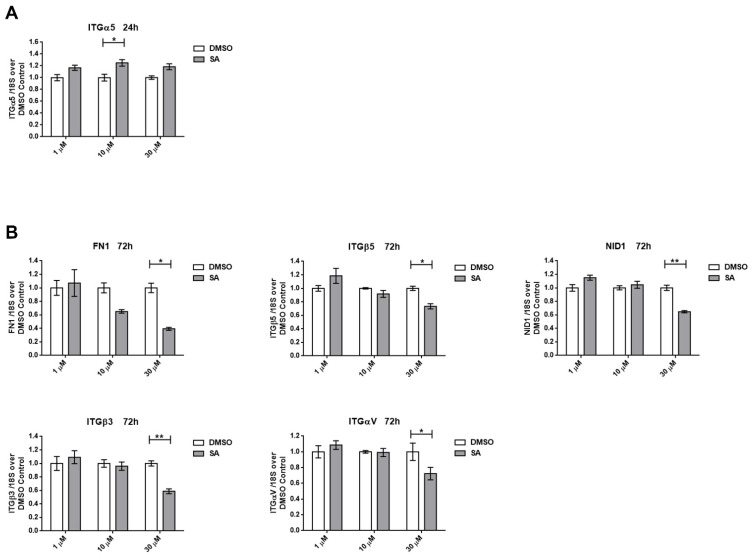
Altered gene expression signature of H1299 cells after SA treatments. A broad panel of genes related to cell adhesion and extracellular matrix composition and remodeling was tested. (**A**) Significant gene expression of control and A549 with SA treated for 24 h. (**B**) Significant gene expression of control and H1299 with SA treated for 72 h. Data represent mean ± SEM gene expression with respect to the 18S-housekeeping gene of three different experiments. * *p* < 0.05, ** *p* < 0.01.

Although SA effects over migration were more prominent in H1299 cell line, it seems that transcriptomic changes are lower at 24 h. We only detected a significant upregulation in the expression of the ITGα5 gene (Figure 7A). When we evaluated gene expression in samples after 72 h of SA treatment, we observed a decreased expression of ECM members (FN1 and NID1) and adhesion molecules (ITGαV, ITGβ3 and ITGβ5), mainly at 30 µM (Figure 7B). Other genes related to the ECM or cell–ECM interaction were assayed, although no significant expression changes were detected either at 24 h, or 72 h of SA treatment (Figure S5). Finally, SCD expression was also upregulated in H1299 cells after 72 h of SA treatments (Figure S6A,B).

Our results showed that SA modified the expression of genes related to cell adhesion, ECM compounds and ECM remodeling which are directly or indirectly associated with EMT, and they could suggest a reversion of EMT as a result of SA treatments. We have evaluated different canonical EMT genes and we found increased expression of mesenchymal markers (ZEB1, TWIST1) after 24 h of treatment (Figure S5A), and reduction of the epithelial CDH1 gene and its ratio over CDH2 after 72 h of SA treatment in A549 cells (Figure S5B). On the other hand, H1299 did not show altered expression of EMT genes after 24 h (Figure S6A). H1299 cell lines showed ACTA2 mesenchymal gene reduced expression, while the ratio CDH1/CDH2 was upregulated after 72 h of SA treatment (Figure S6B). All the other evaluated EMT genes (ZEB1, ZEB2, TWIST1, TWIST2, TGFβ1, CDH, CDH2 VIM and SNAIL) did not show altered expression after treatment in both cell lines (Figures S5AB and S6A,B).

## 4. Discussion

Although the antitumoral effects of lipid metabolism inhibition have been widely demonstrated, the effects of SA over cancer cell lines have not been shown until now. Sterculic acid is a lipid described as a stearoyl–CoA desaturase inhibitor, so this molecule could be used as a new lipid metabolism modulator to control de novo lipid synthesis. SCD inhibition, or silencing, reduces cell proliferation in A549 cells [37], whereas it promotes Caspase-3-dependent apoptosis in H1299 cells [38]. Furthermore, it was observed that SCD1 inhibition induces ER stress and the subsequent cell death [39]. Our results demonstrate a low SA cytotoxicity (150–250 µM) in NSCLCs, which is increased as a result of serum starvation. This effect is in concordance with other authors that observed an IC50 reduction of SCD1 inhibitors to promote cell death when they reduced serum levels from 10% to 2% in A549 and H1299 cells [39]. We also observed this effect in the normal bronchial epithelial cell line NL20. Other SCD1 inhibitors with higher affinity for SCD, such as CAY10566, MF-438 or A939572, require much lower concentrations than SA to induce their cytotoxic effects [28]. These data suggest that SA it not a very potent SCD-1 inhibitor, and therefore may induce its different cellular effects independently of SCD1 inhibition. Our results suggest that SA-induced cell-death is associated with apoptosis induction because Caspase-3 cleavage was detected in H1299 cells exposed to high SA doses (100 µM) for a long time. This observation is in concordance with an induced H1299 apoptosis after treatments with the SCD inhibitor CVT-11127 [38]. Caspase-3 cleavage is a central element of the execution-phase of apoptosis, but it can also mediate an alternative pathway activation of pyroptosis [40]. Lipid peroxidation associated to ferroptosis could be an alternative cell-death mechanism, nevertheless Caspase activation has not been described in ferroptosis cell-death [41]. Simultaneous activation of some cell death mechanisms has been already observed [42,43]. We postulate that Caspase-3 activation could be the final step of an alternative/mixed cell-death mechanism, although pyroptosis could not be excluded as an alternative SA-induced cell-death mechanism.

Previously, we performed a genome-wide transcriptome analysis in retinal cells and we observed that many cell pathways related to extracellular matrix, cell adhesion and actin–cytoskeleton reorganization altered after SA treatment [35]. Furthermore, an SCD1-independent change was observed in the expression of adhesion and ECM genes such as ITGα5, Col1a1, CAV1 [35]. In that work we also detected altered expression of other genes related to: collagen (COL1A1, COL1A2, COL3A1, COL5A2, COL7A1, COL8A1, COL9A1, COl11A1, COL16A1, COL17A1), laminins (LAMA3, LAMC1), thrombospondins (THBS3), fibronectin (FN1), versican (VCAM), cell adhesion (ITGB2, NCAM1, SDC2, CLDN16, CDH1, CDH3, CDH10, CDH15), growth factors (IGFBP3, IGFBP4, IGFBP5, IGFBP8, IGFBP9) and actin-cytoskeleton reorganization (Filamin, Parvin, MLC, MLCP and RHO) [35]. Aramchol, another SCD1 inhibitor, has been recently described as presenting antifibrotic activity independently of SCD1 expression. In hepatic stellate cells (HSCs), Aramchol down-regulated ACTA2, COL1a1 and MMP2 expression as well as collagen secretion [44].

Functional studies of cell migration, such as wound healing and Boyden chamber assay, showed that SA treatments reduce tumor cells migration. We also observed that this effect is more prominent in the H1299 cell line. In order to understand the SA-induced migratory modulation, we evaluated the effects of SA treatment over genes related to cell migration and extracellular matrix. Since the baseline expression signature of both cell lines is different, up and down-regulated genes differ between both cell lines [45,46,47], highlighting the relevance of the genetic background of the cell. For instance, we could not detect expression of TIMP3 and IL-6 in H1299 cells, while both genes presented SA-altered expression in the A549 cell line. CD44 is another example because it is modulated by SA (possibly through p53 signaling) in A549 cells while it is not changed in the H1299 cell line (a TP53 deficient cell line) [48]. However, our data showed that the H1299 cell line presented more a prominent migratory response to SA treatments, while A549 cell lines showed the opposite effect (an intense regulation of genes but lower migratory modulation after SA treatments).

Many other genes presented altered gene expression upon SA treatments. For example, we observed that FN1 gene is slightly down-regulated after 24 h, and it is strongly reduced after 72 h in both cell lines. FN is a glycoprotein involved in cell adhesion, cell motility, wound healing and maintenance of cell shape, but it also participates in the deposition of other ECM compounds [49,50]. Fibronectin has been described as a classical mesenchymal marker [51] and it is up-regulated in many tumors [52,53]. Therefore, the reduced FN expression could contribute to the decreased migration observed after treatment. Incidentally, reduced cell migration has been observed after FN1 genetic depletion in MCF10CA1h [54]. This FN1 down-regulation was also observed in SA-treated retina cells [35].

Fibronectin interacts with α5β1 integrin to promote cell proliferation in an MAPK/ERK- and EGF-dependent manner [55], but it also promotes EMT in a STAT3-dependent manner [56]. However, our data does not show a solidly altered expression of α5 integrin or β1 integrin after SA treatments. Fibronectin also interacts with other integrin or adhesion molecules [50]. For instance, integrin αVβ3, fibronectin and fibrin form a ternary complex to control invadopodia formation and promote cell proliferation and metastasis [57]. Previously, we demonstrated that β3 functional blocking reduces invadopodia formation in A549 and H1299 cells, while TGF-β increases invadopodia formation, MMP2 protein and Smad2/3 signaling in H157 NSCLC cells [58]. These data are in concordance with our reduced cell migration and β3 downregulation after SA treatment in H1299 cell line. Furthermore, TGF-β up-regulates β3 integrin expression in NSCLC to promote lymph node metastasis [59] and promotes EMT [60,61]. Combined targeting of TGF-β and β3 integrin reduces metastasis [59,60]. Recently, it has been proposed that αVβ3 integrin also induces EMT in a TGF-β-independent manner in A549 cells. This partial EMT induction promotes cell motility but it does not require E-cadherin (CDH1) absence [62]. This EMT is linked to ZEB1 and it is inhibited by miR200, which suppresses membrane location of αVβ3 without effects over αV or β3 integrin intracellular expression [62]. These multiple mechanisms of EMT induction could explain the bi-phasic expression of β3 integrin in A549 cells. This integrin is up-regulated at low SA doses at 24 h, while it is down-regulated at high doses and longer times of treatment. Integrin β3 is closely related to MMPs [63], TIMPs [64] and other ECM remodeling mechanisms [65,66]. TIMP3 is an ADAMs and MMPs inhibitor with tumor suppressor activity. This gene is an inhibitor of angiogenesis and TNF-α, but it is also a component of ECM [67] which is associated with cell migration and ECM remodeling in A549 cells and primary NSCLC patients [68,69]. TIMP3 presents an opposite relation with ITGβ3, because it inhibits FAK and β3 recruitment to focal adhesions [67]. All these data would be in concordance with our observation of β3 integrin reduction in A549 cells, and the reduction in cell migration.

αV and β5 integrins are adhesion molecules related to cancer progression and tumor cell migration and invasion [70]. In concordance with the reduced H1299 migration, we detected low expression of both integrins in H1299 after SA treatment. Reduced migration was also observed in breast cancer cells after αV integrin silencing, while integrin overexpression conferred stem-cell-like properties [71]. However, we detected an intense αV and β5 integrin overexpression in SA-treated A549 cells. This result could justify the absence of reduced wound healing assay of A549. Nevertheless, we cannot exclude the possibility of activation of compensatory mechanisms to overcome FN1 deficiency or reduced integrin adhesion/signaling. Bidirectional cell–ECM control has been demonstrated [72], for instance, ZEB1 knockdown up-regulates laminin and β4 integrin and down-regulates α3 and β1 integrin [73], or compensatory signaling mechanisms were observed after β3 integrin silencing [74,75].

NID1 is a basement membrane with prometastasic characteristics, which it is also up-regulated in the lung metastasis of breast cancer cells. Its expression correlates with poor outcomes [76]. miR-767-3p reduces NID1 expression and inactivates the NID1/PI3K/Akt/EMT pathway to reduce tumor growth in NSCLC [77]. NID1 down-regulation was detected after 30 μM SA treatment in H1299 cells, while the A549 cell line did not modify its NID1 expression. This expression pattern is in agreement with our wound healing results.

CD44 is the main cell surface receptor of hyaluronan (HA), which is associated with the rapid remodeling of the matrix [48]. CD44 is a classic cancer stem cell marker [78] and it is closed related to TGF- β-induced EMT in the A549 cell line [79]. We detected a strong inhibition of CD44 expression in the A549 cell line after 72 h of SA treatment, while H1299 kept constant levels of the gene. Since H1299 is a null TP53 cell line and P53 is a CD44 repressor [48], the absence of response to SA treatment was not surprising in this cell context.

IL-6 is a cytokine that has been related to tumor metastasis and EMT through STAT3 signaling [80]. SA has been demonstrated to reduce 7KCh-induced IL-6 levels in retinal cells in a TLR4 and ER-Stress mediated process [32], although SA alone did not alter IL-6 levels [35]. We could not detect IL-6 expression in H1299 cells in concordance with Bihl and co-workers [81]. Molecules with NF-Κβ inhibitory activity reduce IL-6 expression in a STAT3 dependent manner in A549 cells, and they also reduce cell migration, invasion and alter EMT markers [82,83,84]. Furthermore, TLR4 knockdown reduces IL-6 levels and cell migration of A549 cell by Pi3K/AKT pathway inhibition [85]. Our results showed an increased expression of IL6 at 24 h and this may explain the lack of effect of SA in wound healing assay, while the decreased levels of IL6 in SA-treated cells at 72 h reduced wound healing closure. A positive feedback, called IL6 amplifier, associated with the NF-Κβ-IL-6-STAT3 axis in non-immune cells has been described [86]. IL-6 binding to its receptor (IL-6R) depends on ADAM17 because it forms a soluble receptor (sIL-6Rα) necessary for the pathway signaling [87]. Since TIMP3 is an ADAM17 inhibitor [88], TIPM3 up-regulation in SA-treated cells could be responsible of IL-6 mRNA reduction in A549 SA-treated cells.

SCD up-regulation after SA treatments was observed in both NSCLC cell lines, which had not been observed in our studies in non-tumorigenic MRPE cells [35]. SCD expression is modulated mainly by SREBP-1 and ChREBP [28,31], although many other pathways can also promote its expression, PPARs; LXR, C/EBP-α, NF-Y, SP-1, β-Catenin, MAPK pathway [31], but some of these factors also downregulate SCD expression [89]. We cannot discard the possibility of a SCD1 overexpression as a result of cellular response to reduced levels of fatty acids (FA). Oleic acid restores the migration of SCD-inhibited breast cancer cell [90] and it is a PTEM/AKT-dependent process in colorectal cancer [91]. However, oleic acid supplementation does not restore the reduced GSK3b/AKT signaling in SCD1-silenced A549 cells [37].

Cell migration and extracellular matrix remodeling are processes closely related to the epithelial–mesenchymal transition. This is a cellular de-differentiation process associated with cancer metastasis, but it is also a normal process during embryonic development and wound healing [92]. Cell adhesion, matrix compounds or cell-microenvironment interaction genes also adapt their expression to the new cellular program and environment [93,94]. Our results show that SA modified the expression of genes related to cell adhesion, the ECM and ECM remodeling, and many of these genes are associated with EMT. We have studied the expression levels broad panel transcription factors which are associated with a mesenchymal phenotype (ZEB1, ZEB2, TWIST1, TWIST2, TGFβ1, CDH2 VIM and SNAIL), as well as the CDH1 epithelial marker. If SA treatment could induce EMT reversion we would expect the increased expression of epithelial markers and reduction of mesenchymal markers. Our results showed that only ACTA2 gene presented a significant reduction of expression in H1299 cell line after 72 h of SA treatment, which is in concordance with Bhattacharya and co-workers that observed ACTA2 gene was downregulated after SCD1-inhibition with Aramchol [44]. Furthermore, SCD1 deficiency has been described to reduce EMT signaling in a GSK3β-dependent manner and promote an epithelial phenotype [95]. Nevertheless, we observed a significant reduced expression of the CDH1 epithelial marker in SA-treated A549 cells. This result is in concordance with our transcriptomic analysis in mRPE cells [35]. This reduction in CDH1 is consistent with the overexpression of its repressors ZEB1 and ZEB2 [96]. SNAIL also showed a non-significant reduced expression in both cell lines, but we think that this does not compensate for the significant (ZEB1, TWIST1) and non-significant (VIM, CDH2, TWIST2) overexpression of some other mesenchymal markers, which in some cases are downstream elements of the SNAIL signal pathway. As summary, we can exclude that SA promotes a reversion to an epithelial phenotype through the classical alteration in mesenchymal markers. On the contrary, SA induces a reduction in lung cancer cells migration capability as a result of modification in the cell–ECM interaction and compounds.

Intratumoral heterogeneity, subpopulations in cell lines or differential cellular response to treatments could be factors associated with drug-tolerant persistent cells or minimal residual disease (MRD). Detection of robust targets or biomarkers would help to overcome these problems, which are the origin of the poor prognosis and the worse evolution of patients. Single-cell technology and microfluidic devices are new approaches to detect markers or predict a cancer’s evolution [97,98,99]. Analysis of SA-treated cell with these methodologies could help to detect key targets/elements with altered expression in a cellular subpopulation that could be masked by its expression in other cellular subpopulations after treatments. These new methodologies would lead us to develop personalized treatments or predict the evolution and/or response of patients in order to obtain therapies with lower secondary effects as well as more efficient and cheaper.

## 5. Conclusions

In this work we report that SA presents interesting properties over cancer cells. We detected an induced cell cytotoxic effect at high SA doses, while we observed cell motility defects as a result of low SA doses. Molecular analysis revealed a transcriptomic signature switch associated with a reduced migratory capacity, which could be also compatible with ECM remodeling failure. Taken together, our results reveal that SA is a natural lipid with migration inhibitory properties, as a consequence of the altered expression of the ECM and adhesion molecules, which may serve as a co-adjuvant in cancer treatments.

## Figures and Tables

**Table 1:** List of primers used in this study.

Gene Name	Oligonucleotide Sequence
ITGβ1-Fow	AACGGGGTGAATGGAACAGG
ITGβ1-Rev	ACTTCCTCCGTAAAGCCCAG
ITGβ3-Fow	TTGATGCTTATGGGAAAATCCG
ITGβ3-Rev	ACCTTGGCCTCAATGCTGAA
ITGβ5-Fow	CAAACTCGCGGAGGAGATGA
ITGβ5-Rev	AATGCACGGATTGGTCTGGT
ITGα5-Fow	TGGCCTTCGGTTTACAGTCC
ITGα5-Rev	GGAGAGCCGAAAGGAAACCA
IL-6-Fow	TACCCCCAGGAGAAGATTCC
Il-6-Rev	TTTTCTGCCAGTGCCTCTTT
ITGαV-Fow	CCAAAGCAAACACCACCCAG
ITGαV-Rev	GCTCCAAACCACTGATGGGA
TIMP3-Fow	CAAGGGGCTGAACTATCGGT
TIMP3-Rev	TCGGTCCAGAGACACTCGTT
VIM-Fow	CAGGACTCGGTGGACTTCTC
VIM-Rev	TAGTTGGCGAAGCGGTCATT
SNAIL-Fow	CTATGCCGCGCTCTTTCCTC
SNAIL-Rev	GTAGGGCTGCTGGAAGGTAAA
TGFβ1-Fow	TTGAGCCGTGGAGGGGAAAT
TGFβ1-Rev	GCGTTGATGTCCACTTGCAG
TWIST1-Fow	ATTCAGACCCTCAAGCTGGC
TWIST1-Rev	CATCCTCCAGACCGAGAAGG
TWIST2-Fow	AGCAAGAAGTCGAGCGAAGA
TWIST2-Rev	CTTGTCAGAGGGCAGCGT
ZEB1-Fow	ACGCTTTTCCCATTCTGGCT
ZEB1-Rev	TTTGCCGTATCTGTGGTCGT
ZEB2-Fow	CCAAGGAGCAGGTAATCGCA
ZEB2-Rev	GTGCGAACTGTAGGAACCAGA
ACTA2-Fow	CCAACTGGGACGACATGGAA
ACTA2-Rev	CAGGGTGGGATGCTCTTCAG
CDH1-Fow	GACGCGGACGATGATGTGAA
CDH1-Rev	GAAACTCTCTCGGTCCAGCC
CDH2-Fow	GCCCAAGACAAAGAGACCCA
CDH2-Rev	ACCCAGTCTCTCTTCTGCCT
FN1-Fow	TTCCAAGCACAGCCACTTC
FN1-Rev	AACTCTGCTCCCCATCCTCA
NID1-Fow	ACGGGGATGACTTCGTCTCT
NID1-Rev	GGGGGTTCACTCGTAGCAAT
CD44-Fow	GACATCTACCCCAGCAACCC
CD44-Rev	CTGTCTGTGCTGTCGGTGAT
18S-Fow	ATGCTCTTAGCTGAGTGTCCCG
18S-Rev	ATTCCTAGCTGCGGTATCCAGG

## Data Availability

Not applicable.

## Supplementary Materials

The following are available online at https://www.mdpi.com/article/10.3390/cancers13174370/s1, Figure S1: Sterculic acid induces cell death at high dose in lung cancer cells cultured in 10% serum-supplemented medium. Figure S2: Sterculic acid induces cell death at high dose in non-tumorigenic human bronchial epithelial cell line NL20. Figure S3. A549 Genes whose expression was not modified after SA treatments. Figure S4. H1299 Genes whose expression was not modified after SA treatments. Figure S5. Expression of a broad panel of genes associated to EMT in A549 after SA treatments. Figure S6. Expression of a broad panel of genes associated to EMT in H1299 after SA treatments. Figure S7. Original western-blots.
