# Supplementary material for: Sterculic Acid Alters Adhesion Molecules Expression and Extracellular Matrix Compounds to Regulate Migration of Lung Cancer Cells
Source: Cancers (Basel). 2021 Aug 29;13(17):4370. doi: 10.3390/cancers13174370 (PMC8431022; doi:10.3390/cancers13174370)
Supplement: Supplementary file 1 [file cancers-13-04370-s001.zip › cancers-1301186-supplementary.pdf]

cancers

MDPI

Supplementary Materials:

Sterculic Acid Alters Adhesion Molecules Expression and Extracellular Matrix Compounds to Regulate Migration of Lung Cancer Cells

Rafael Pelaez, Rodrigo Ochoa, Ana Pariente, Angela Villanueva-Martínez, Álvaro Pérez-Sala and Ignacio M. Larráyoz

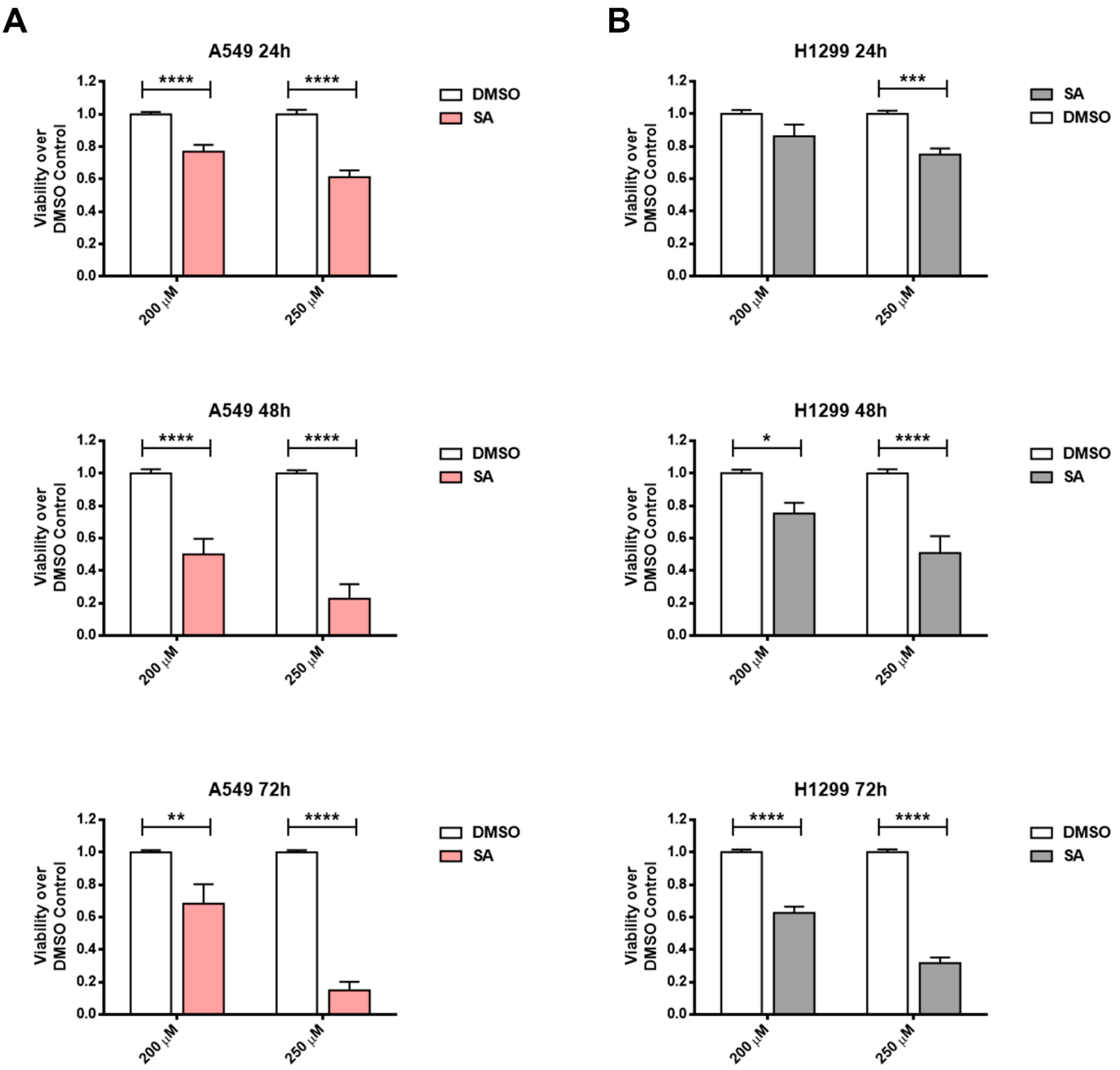

Figure S1. Sterculic acid induces cell death at high dose in lung cancer cells cultured in 10% serum-supplemented medium. Cell viability after SA treatment (200–250 µM) was measured by MTS method. (A) Cell viability over DMSO control in A549 cells. White bars correspond to cells treated with vehicle (DMSO) while pink bars correspond to cells treated with SA (200–250 µM). (B). Cell viability over DMSO control of H1299 cells. White bars correspond to cells treated vehicle (DMSO) while grey bars correspond to cells treated with SA (200–250 µM). Data represented mean ± SEM of at least three different experiments. * $p < 0.05$, ** $p < 0.01$, *** $p < 0.001$, **** $p < 0.0001$.

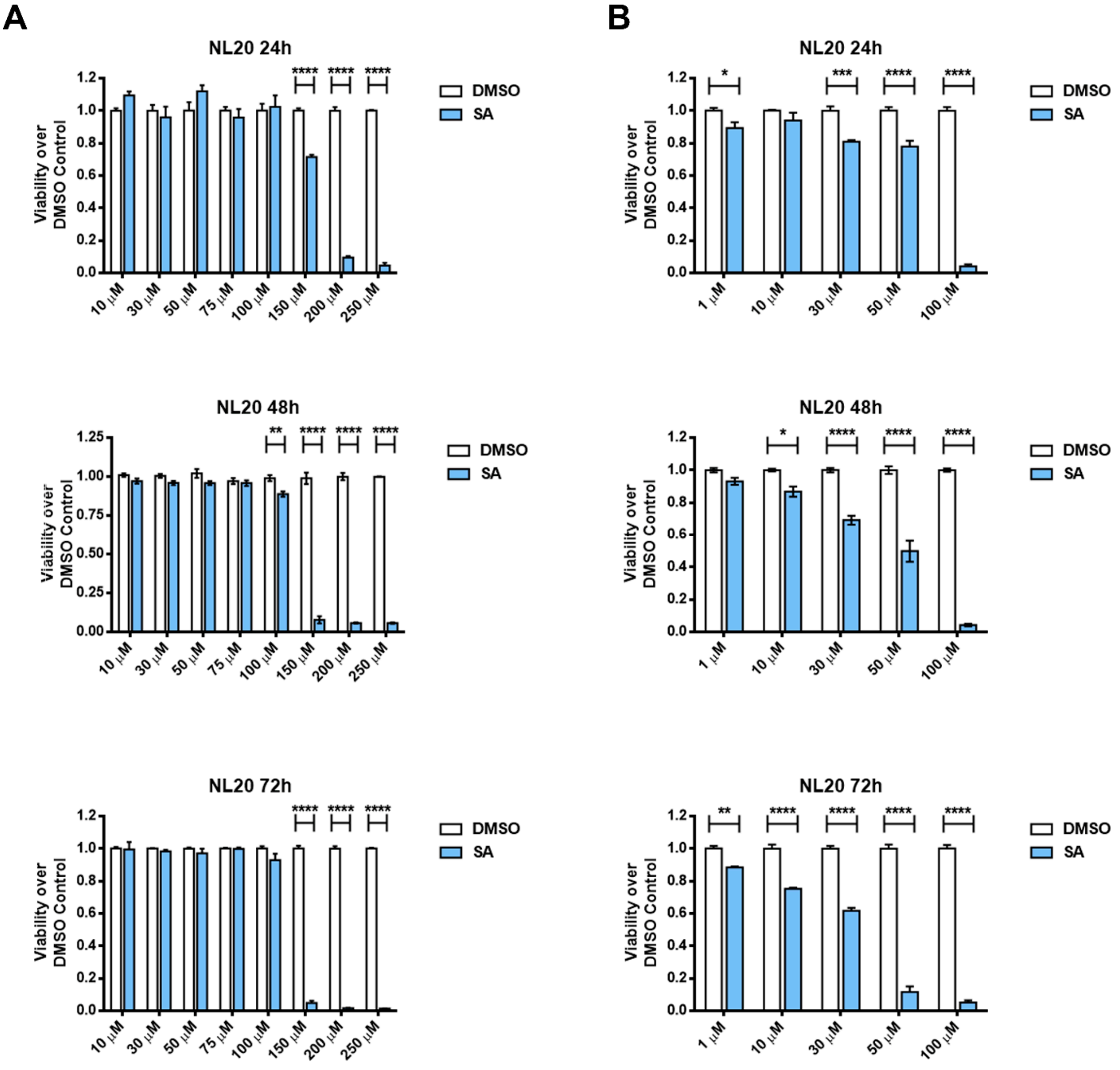

Figure S2. Sterculic acid induces cell death at high dose in non-tumorigenic human bronchial epithelial cell line NL20**.** Cell viability after SA treatment was measured by MTS method. (A) Cell viability over DMSO control in NL20 cells in 10% serum supplemented medium after 24, 48 and 72 hours of treatment. White bars correspond to cells treated with vehicle (DMSO) while blue bars correspond to cells treated with SA (10–250 μM). (B) Cell viability over DMSO control in NL20 cells in serum free medium after 24, 48 and 72 hours of treatment. White bars correspond to cells treated with vehicle (DMSO) while blue bars correspond to cells treated with SA (1–100 μM). Data represented mean ± SEM of one experiment * $p < 0.05$, ** $p < 0.01$, *** $p < 0.001$, **** $p < 0.0001$.

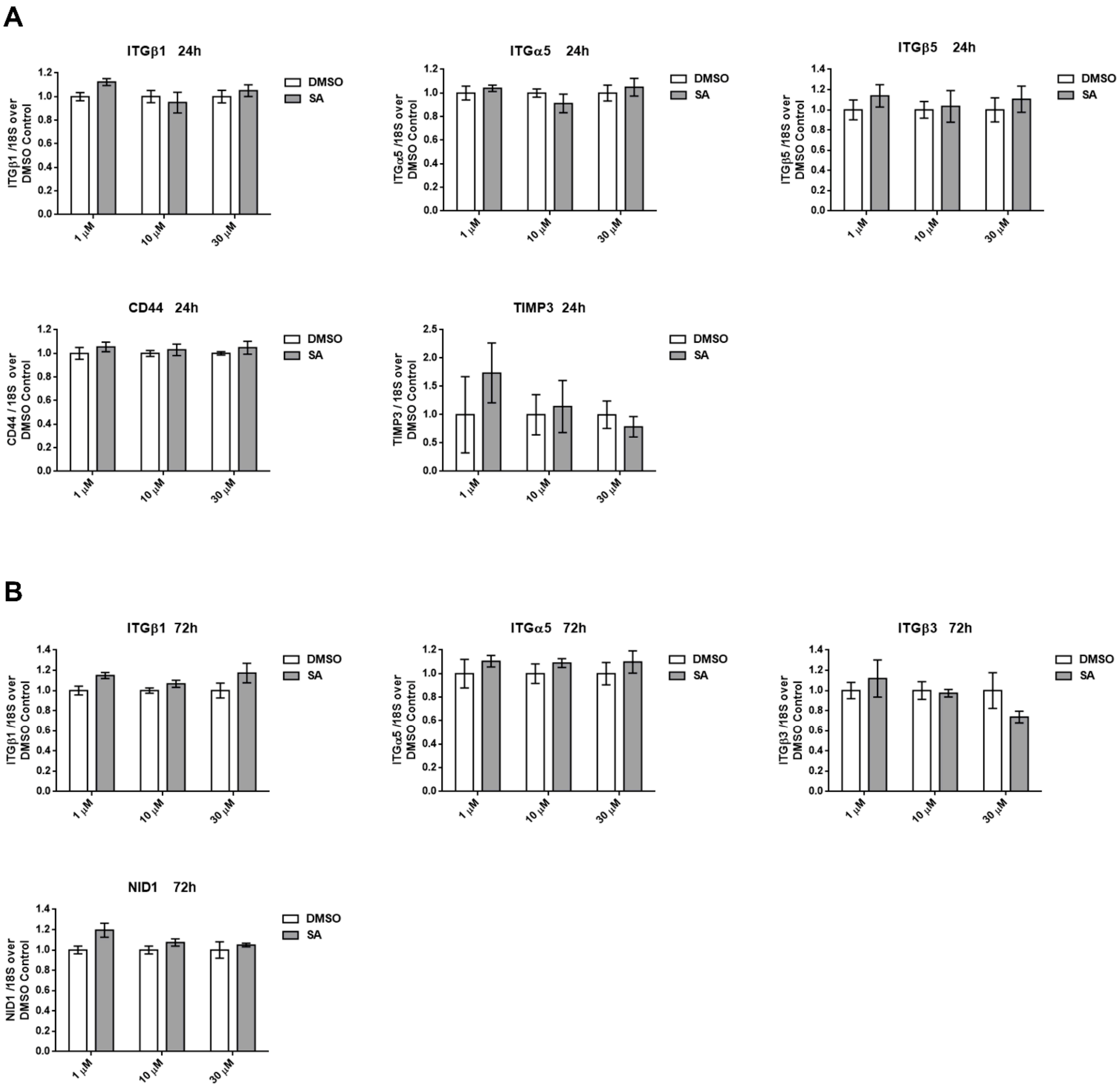

Figure S3. A549 Genes whose expression was not modified after SA treatments. (A) Non-significant altered expression of control and A549 with SA treated for 24 hours (B) Non- significant altered expression of control and A549 with SA treated for 72 hours. Data represent mean ± SEM gene expression with respect to the 18S-housekeeping gene of three different experiments.

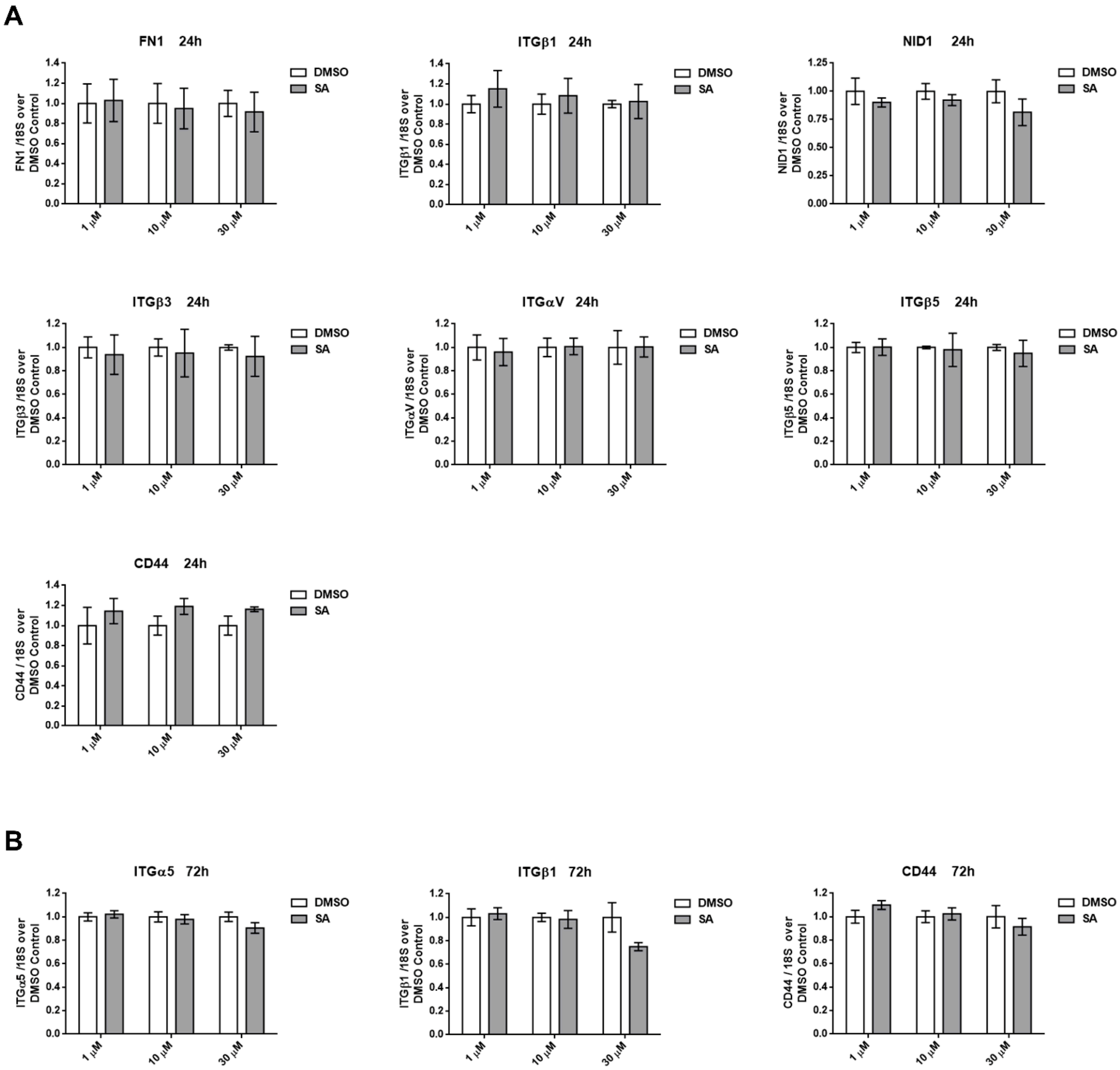

Figure S4. H1299 Genes whose expression was not modified after SA treatments. (A) Non-significant altered expression of control and H1299 with SA treated for 24 hours (B) Non-significant altered expression of control and H1299 with SA treated for 72 hours. Data represent mean ± SEM gene expression with respect to the 18S-housekeeping gene of three different experiments.

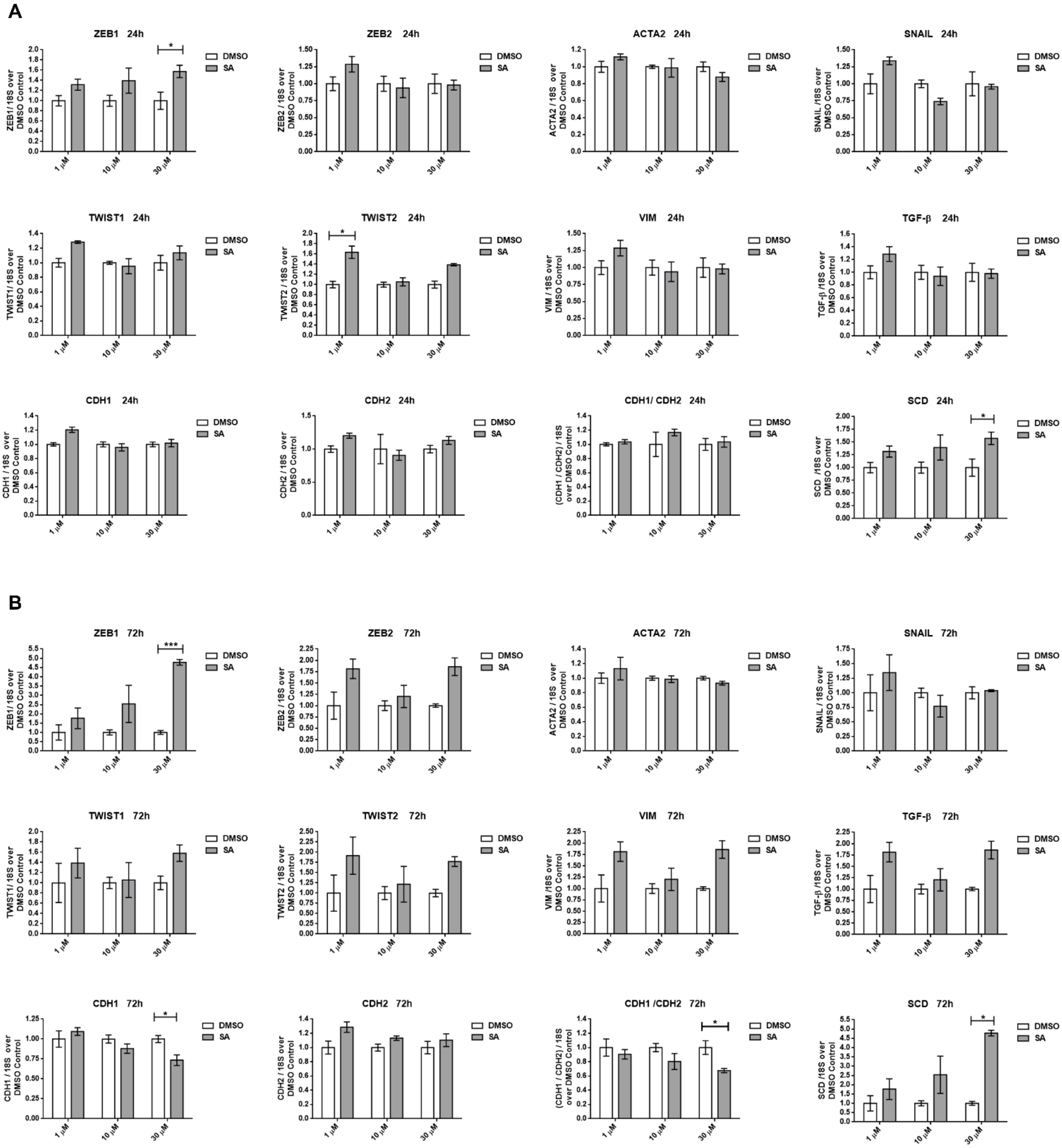

Figure S5. Expression of a broad panel of genes associated to EMT in A549 after SA treatments. (A) Gene expression of control and SA treated cells for 24 hours (B) Gene expression of control and SA treated cells for 72 hours. Data represent mean ± SEM gene expression with respect to the 18S-housekeeping gene of three different experiments. * $p < 0.05$, *** $p < 0.001$.

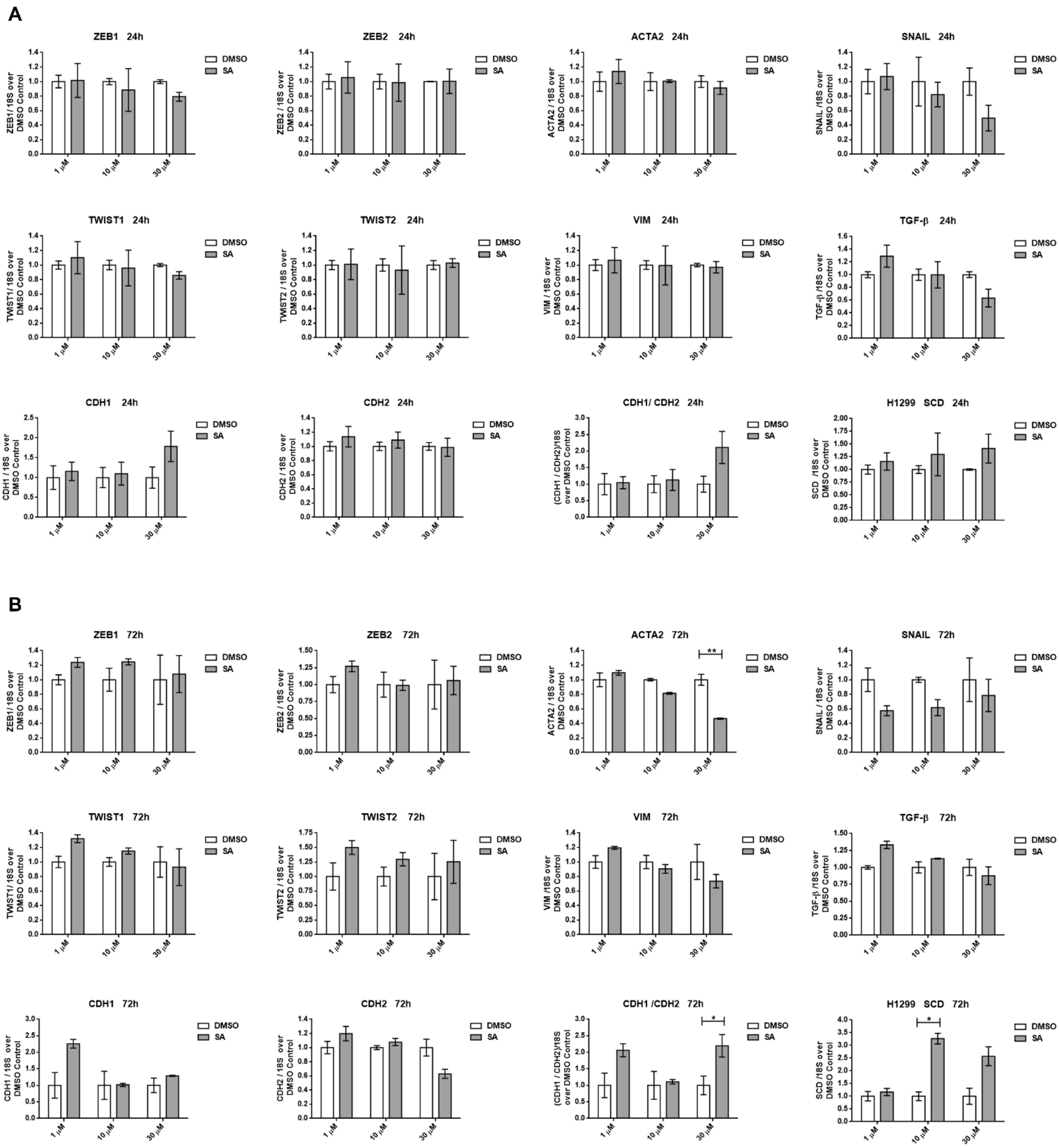

Figure S6. Expression of a broad panel of genes associated to EMT in H1299 after SA treatments. (A) Gene expression of control and SA treated cells for 24 hours (B) Gene expression of control and SA treated cells for 72 hours. Data represent mean ± SEM gene expression with respect to the 18S-housekeeping gene of three different experiments. * $p < 0.05$, ** $p < 0.01$.

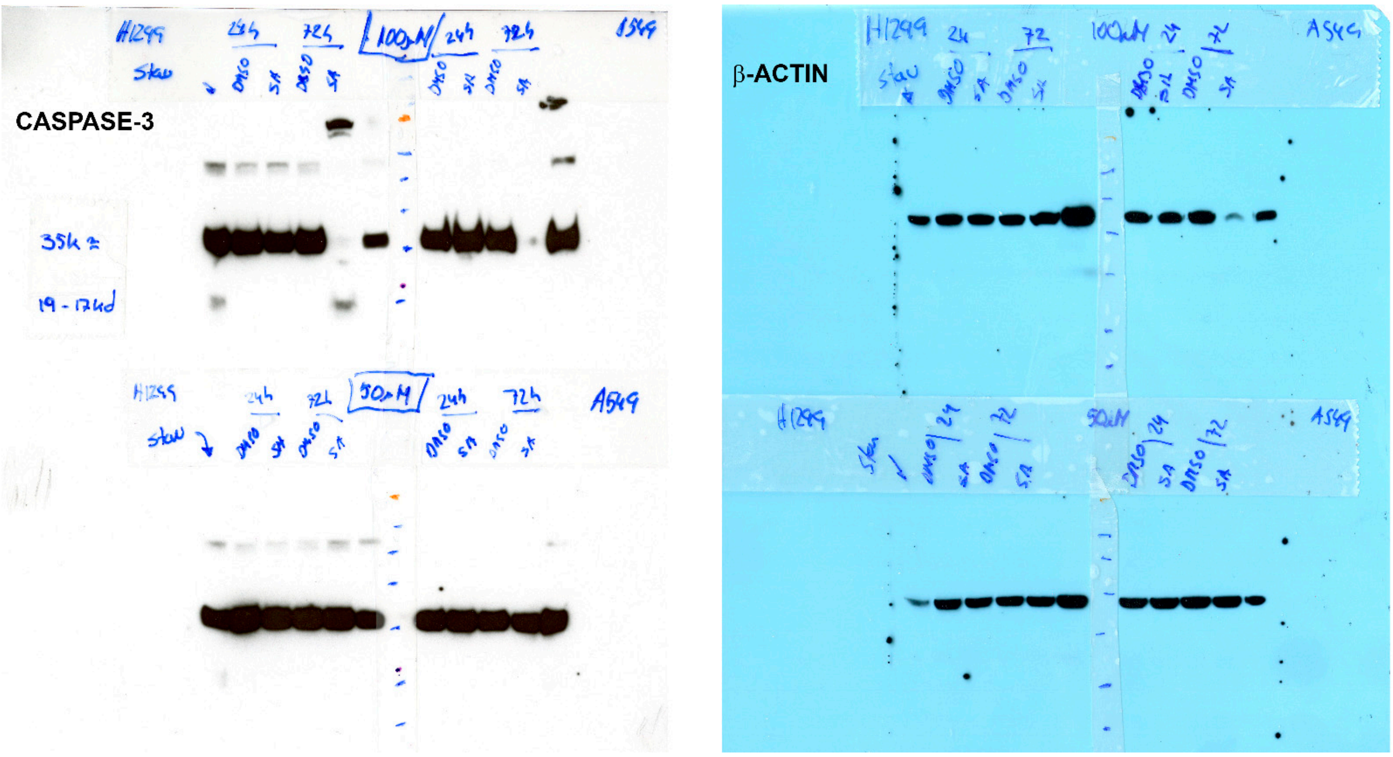

Figure S7. Original Western blots .
